# Eosinophilic Esophagitis and Risk of Non-Alcoholic Fatty Liver Disease and Cirrhosis: A Multi-Center Retrospective Study

**DOI:** 10.7150/ijms.123768

**Published:** 2026-07-13

**Authors:** Shuo-Yan Gau, Yu-Jung Su, Shih-Chi Yang, Chih-Lung Wu, Solomon Chih-Cheng Chen, Hui-Chin Chang, Meng-Che Wu

**Affiliations:** 1 Department of Medical Education, Ditmanson Medical Foundation Chia-Yi Christian Hospital, Chiayi City, Taiwan.; 2 Institute of Allergology, Charité-Universitätsmedizin Berlin, Corporate Member of Freie Universität Berlin and Humboldt-Universität zu Berlin, Berlin, Germany.; 3 Department and Graduate Institute of Business Administration, National Taiwan University, Taipei, Taiwan.; 4 Orthopedics Department, Chi-Mei Medical Center, Tainan, Taiwan.; 5 Education Center, National Cheng Kung University Hospital, College of Medicine, National Cheng Kung University, Tainan, Taiwan.; 6 Department of Orthopedic Surgery, Chung Shan Medical University Hospital, Taichung, Taiwan.; 7 School of Medicine, Chung Shan Medical University, Taichung, Taiwan.; 8 Department of Pediatrics, Ditmanson Medical Foundation Chia-Yi Christian Hospital, Chiayi City 600566, Taiwan; 9 Department of Pediatrics, College of Medicine, Taipei Medical University, Taipei 110301, Taiwan.; 10 Evidence-based Medicine Center, Chung Shan Medical University Hospital, Taichung, Taiwan.; 11 Library, Chung Shan Medical University Hospital, Taichung, Taiwan.; 12 Division of Pediatric Gastroenterology, Children's Medical Center, Taichung Veterans General Hospital, Taichung, Taiwan.; 13 Department of Post-Baccalaureate Medicine, College of Medicine, National Chung Hsing University, Taichung, Taiwan.

## Abstract

**Background and Aim:**

Eosinophilic esophagitis (EoE) is a chronic Th2-mediated inflammatory disease. Given its systemic immune activation and eosinophil-rich pathology, this study aimed to evaluate the longitudinal risk of non-alcoholic fatty liver disease (NAFLD) and cirrhosis among patients with EoE.

**Methods:**

A retrospective cohort study was conducted using the TriNetX US Collaborative Network. Adults (≥18 years) with an EoE diagnosis were matched 1:1 with EoE-free controls based on demographics, comorbidities, and healthcare utilization. Patients with prior liver disease or malignancy were excluded. Incident NAFLD and cirrhosis were evaluated excluding diagnoses within 3 months of the index date. Sensitivity analyses explored alternative EoE definitions (proton pump inhibitor use, topical corticosteroids, endoscopy records) and longer wash-out periods.

**Results:**

Compared with matched controls, EoE patients had a significantly elevated risk of NAFLD (HR = 1.409, 95% CI: 1.277-1.555) and cirrhosis (HR = 1.529, 95% CI: 1.176-1.988). Elevated risks persisted using PPI-based (NAFLD: HR = 1.705, 95% CI: 1.535-1.894; cirrhosis: HR = 1.962, 95% CI: 1.478-2.605), steroid-based (NAFLD: HR = 1.746, 95% CI: 1.561-1.953; cirrhosis: HR = 2.101, 95% CI: 1.557-2.837), and endoscopy-based definitions (NAFLD: HR = 1.951, 95% CI: 1.506-2.528).

**Conclusions:**

EoE is associated with higher long-term risks of NAFLD and cirrhosis. These findings underscore the importance of hepatic surveillance and immune-metabolic research in EoE patients. However, potential residual confounder caused by comedications should be cautiously interpreted.

## Introduction

Eosinophilic esophagitis (EoE) is a chronic immune-mediated inflammatory condition of the esophagus, characterized clinically by symptoms of dysphagia, food impaction, and feeding difficulties, and histologically by eosinophil-predominant infiltration of the esophageal mucosa^1^. EoE affects an estimated 50-60 per 100,000 U.S. adults^2^, and is increasingly viewed as a systemic allergic disorder driven by type-2 immunity^3^. Treatment for eosinophilic esophagitis (EoE) involves several approaches, including proton pump inhibitors (PPIs), topical corticosteroids, dietary modifications, and esophageal dilation^4^. PPIs are well-tolerated and effective in around 41% of patients. Topical corticosteroids, such as fluticasone and budesonide, are associated with histologic remission in nearly 65% of cases^5^. EoE impairs patients' quality of life and incurs significant healthcare costs from repeated endoscopies and ongoing therapy^6^.

Non-alcoholic fatty liver disease (NAFLD) represents a spectrum ranging from simple steatosis to non-alcoholic steatohepatitis (NASH), the latter characterized by hepatocellular injury, inflammation, and progressive fibrosis that can culminate in cirrhosis and hepatocellular carcinoma, and afflicts more than 25% of the global population^7^. It has become a predominant indication for liver transplantation and cirrhosis-related morbidity. However, beyond these metabolic drivers, there is growing appreciation for the role of immunologic and inflammatory pathways in NAFLD progression. Chronic low-grade inflammation originating from adipose tissue and the gut, as well as activation of innate and adaptive immune cells in the liver, contribute to the transition from benign steatosis to NASH and fibrosis^8^. Epidemiological studies also reported association between NAFLD and various inflammatory comorbidities^9^, ^10^. Eosinophils, the signature cells in EoE, are an important source of Th2 cytokines and have been observed to accumulate in fatty livers during NASH progression^11^. These observations raise the question of whether an allergic disorder like EoE might impact the liver through systemic immunologic mechanisms.

Given the shared features such as Th2 cytokine signaling and epithelial barrier defects, EoE was reported to be potentially associated with elevated risk of new-onset hepatic diseases^4^, ^12^. In a recent observational study of US population, it was stated that EoE could be associated with metabolic-dysfunction-associated steatotic liver disease^13^. However, to date, longitudinal study evaluating the association between EoE and liver outcomes was not available. Therefore, we conduct a retrospective cohort study to address the knowledge gap.

## Materials and Methods

**Figure 1:**
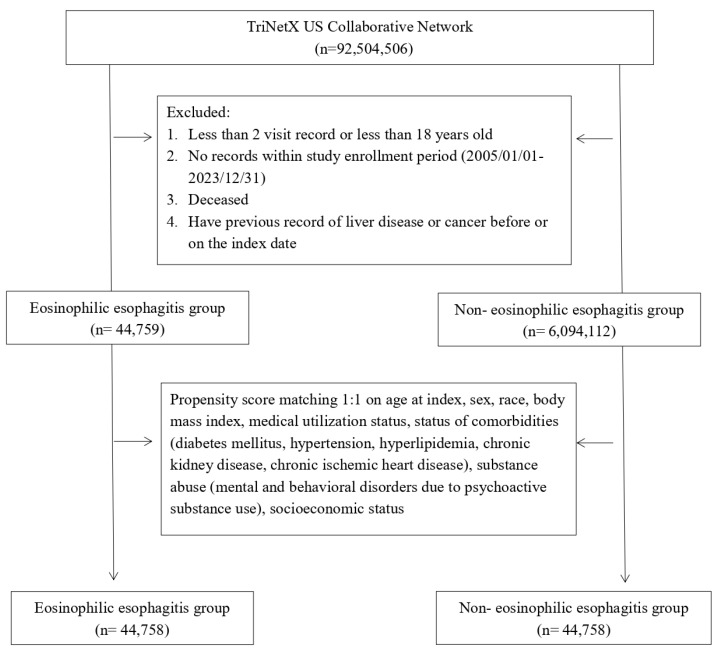
Patient selection flowchart.

This multi-center retrospective cohort investigation was based on an analysis of the TriNetX Research Network, an internationally curated and prospectively updated repository of de-identified electronic health records contributed by partner institutions, which has been widely used in health economics and outcomes research^14^-^16^. Analyses employed the US Collaborative Network subset. Applied diagnostic codes are presented in **Table S1**. Eligible participants were adults (≥18 years) who registered at least two healthcare visits between January 2005 and December 2023 (**Figure 1**). Individuals carrying a diagnostic code for EoE formed the exposure cohort; those with no record of EoE constituted the comparison cohort. Both groups excluded anyone deceased, any patient with a history of neoplasms before or on the index date, or anyone having record of liver disease coded as ICD-10-CM K70-K77 before or on the index date. Propensity-score matching was undertaken for each analysis, and covariate balance was assessed before and after matching. The propensity model incorporated age at index, sex, race, body-mass index, healthcare utilization status, comorbidities (diabetes mellitus, hypertension, hyperlipidemia, chronic kidney disease, chronic ischemic heart disease), psychoactive-substance-related disorders, and socioeconomic status. Primary outcomes were incident NAFLD and newly diagnosed liver fibrosis or cirrhosis. Events occurring within three months of the index date were excluded. To address potential over-matching and reverse causality, multiple alternative analytical strategies featuring distinct matching algorithms and varying wash-out windows were explored in prespecified sensitivity analyses (**Table S2**). All statistical procedures were executed within the TriNetX platform. Baseline balance was calculated using standardized mean differences (SMD), with an SMD > 0.10 indicating notable imbalance. Relative risks of the outcomes of interest were expressed as hazard ratios (HRs) accompanied by 95 % confidence intervals (CIs).

## Results

Prior to propensity score matching, significant differences were observed between the EoE and non-EoE cohorts regarding age, sex, race, body mass index, healthcare utilization, and comorbidity profiles. Specifically, patients with EoE tended to be younger, more likely to be white, male, and had higher rates of ambulatory visit. Following matching, baseline characteristics between the two groups were well balanced, with all standardized mean differences reduced to less than 0.1 (**Table 1**).

**Figure 2:**
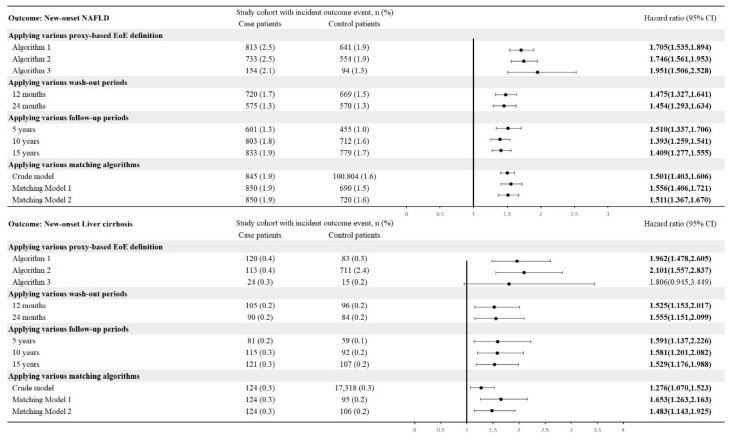
Risk of NAFLD and liver cirrhosis in various sensitivity models. *Legends:* NAFLD, non-alcoholic fatty liver disease; EoE, eosinophilic esophagitis. Detailed information regarding sensitivity model is reported in Table S2.

During a follow-up period of up to 15 years with a 3-month wash-out, patients with EoE exhibited a significantly higher risk of developing NAFLD compared to matched controls (HR=1.409; 95% CI, 1.277-1.555). Similarly, the risk of liver cirrhosis or fibrosis was markedly elevated in the EoE cohort compared to non-EoE individuals (HR=1.529; 95% CI, 1.176-1.988). Sensitivity analyses were conducted to validate the robustness of the primary findings. These included applying alternative proxy definitions for EoE: when restricted to patients with proton pump inhibitor prescriptions, the risk of NAFLD remained elevated (HR=1.705; 95% CI, 1.535-1.894), as did the risk of liver cirrhosis or fibrosis (HR=1.962; 95% CI, 1.478-2.605). Similarly, under the corticosteroid-based definition of EoE, the risk of NAFLD was comparable (HR=1.746; 95% CI, 1.561-1.953), and the risk of liver cirrhosis or fibrosis remained significantly increased (HR=2.101; 95% CI, 1.557-2.837). When the EoE cohort was defined by repeated esophagogastroduodenoscopy procedures, consistent association was observed for NAFLD (HR=1.951; 95% CI, 1.506-2.528). Analyses applying longer wash-out periods (12 months and 24 months) similarly demonstrated stable associations. For example, after a 12-month wash-out, the HR for NAFLD was 1.475 (95% CI, 1.327-1.641), and for liver cirrhosis or fibrosis was 1.525 (95% CI, 1.153-2.017). Using a 24-month wash-out, the HR for NAFLD remained elevated at 1.454 (95% CI, 1.293-1.634), with a corresponding HR of 1.555 (95% CI, 1.151-2.099) for liver cirrhosis or fibrosis. Additionally, analyses stratified by different follow-up durations and different matching strategies revealed consistent results. To further address potential confounding by medications associated with metabolic or hepatotoxic effects, an expanded matching model was constructed incorporating systemic corticosteroids, HMG-CoA reductase inhibitors, and glucagon-like peptide-1 receptor agonists into the propensity score algorithm. After adjustment for these medication classes in addition to demographic and clinical covariates, the association between EoE and NAFLD remained significant (HR = 1.404; 95% CI, 1.290-1.528). The risk of liver cirrhosis also persisted (HR = 1.356; 95% CI, 1.075-1.711) (**Figure 2**).

**Figure 3:**
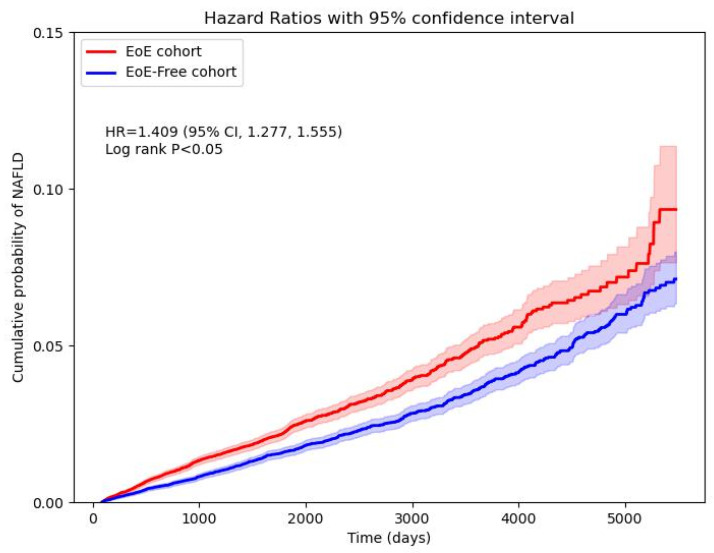
Cumulative probability curve of NAFLD risk in EoE and non-EoE cohorts.

**Figure 4:**
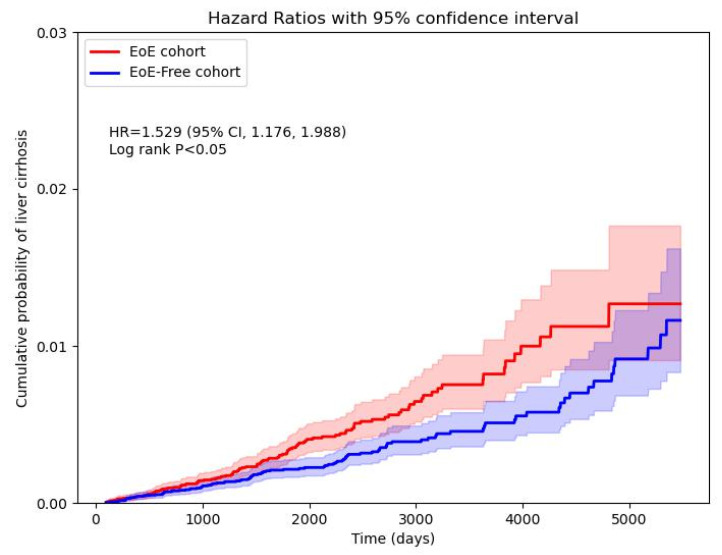
Cumulative probability curve of liver cirrhosis risk in EoE and non-EoE cohorts.

To further evaluate potential medication-related confounding, additional analyses were conducted excluding patients exposed to corticosteroids and, separately, excluding those prescribed proton pump inhibitors. Among EoE patients without corticosteroid exposure, the association with incident NAFLD remained significant (HR = 1.358; 95% CI, 1.135-1.625), whereas the association with liver cirrhosis did not reach statistical significance (HR = 1.277; 95% CI, 0.804-2.029). Similarly, when excluding patients with proton pump inhibitor use, EoE remained associated with NAFLD (HR = 1.242; 95% CI, 1.005-1.534), while the association with cirrhosis was not statistically significant (HR = 1.275; 95% CI, 0.711-2.288) (**Figure 2**). Cumulative probability of NAFLD and liver cirrhosis in EoE and non-EoE cohorts were visualized in** Figure 3** and **Figure 4**.

**Figure 5:**
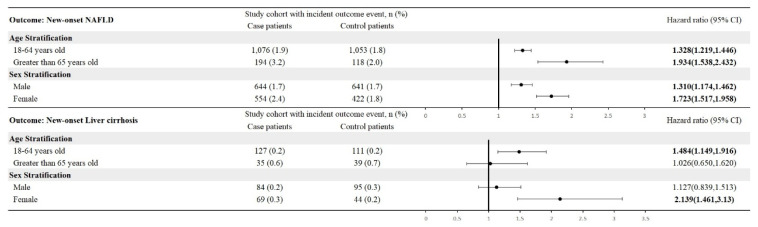
Stratification analysis based on age and sex.

Results of stratification analysis showed that in both male and female sex, the risk of new-onset NAFLD significantly elevated in EoE patients, with the HR of 1.310 (95% CI, 1.174-1.462) and 1.723 (95% CI, 1.517-1.958), respectively. Similarly, in age stratification, the significance of NAFLD risk remained in 18-64 years old subgroup and greater than 65 years old subgroup. As for new-onset liver cirrhosis, female and 18-64 years old EoE patients presented significantly increased risk while comparing with non-EoE controls in the same subgroup (female, HR=2.139, 95% CI, 1.461-3.130; 18-64 years old, HR=1.484, 95% CI, 1.149-1.916). Additional analyses were performed to further evaluate potential confounding by obesity severity and medication exposure. When stratified by BMI category, EoE remained significantly associated with incident NAFLD among individuals with BMI 25-29.9 kg/m² (HR = 1.490; 95% CI, 1.246-1.782), while the association with liver cirrhosis did not reach statistical significance (HR = 1.344; 95% CI, 0.817-2.209). In individuals with BMI ≥30 kg/m², the associations were more pronounced. The risk of NAFLD remained elevated (HR = 1.554; 95% CI, 1.377-1.754), and the risk of liver cirrhosis was significantly increased (HR = 1.994; 95% CI, 1.331-2.987) (**Figure 5**).

## Discussion

In this multi-center retrospective cohort study, we found that patients with eosinophilic esophagitis had a significantly higher risk of developing NAFLD and cirrhosis over time compared to matched controls. To our knowledge, this is the first longitudinal study to demonstrate an association between EoE and adverse hepatic outcomes.

NAFLD represents a spectrum ranging from simple steatosis to non-alcoholic steatohepatitis and progressive fibrosis. Although metabolic dysfunction-associated steatotic liver disease (MASLD) has recently been introduced to replace NAFLD in contemporary consensus statements^17^, the ICD-10 coding structure currently available within the TriNetX platform corresponds to traditional NAFLD-related codes, which was applied in previous real-world studies^18^, ^19^. Therefore, the terminology used in this study reflects the operational definitions embedded within the electronic health record system rather than a conceptual preference. Future studies incorporating updated coding systems may allow more precise alignment with MASLD nomenclature.

Despite theoretical links between EoE and liver disease, data on long-term hepatic outcomes in EoE patients remain limited. To date, most studies investigating a potential association between EoE and NAFLD have been cross-sectional in design. Kohli et al. 13 first reported a potential connection using data from the 2020 National Inpatient Sample in the United States, finding that patients with EoE had approximately 2.4 times higher odds of co-existing NAFLD compared to those without EoE, after adjusting for obesity and other confounders. Our current findings build upon and expand those of the recently published 2024 study by Kohli et al. by demonstrating a similar association in a longitudinal setting. However, there are important methodological differences between our study and the previous literature. Utilizing the 2020 National Inpatient Sample, Kohli et al. captured a very large population (~26 million hospital discharges); however, their study inherently included only patients who had been hospitalized. In contrast, our multi-center cohort was drawn from electronic health records of various medical institutions and included not only inpatient but also outpatient records. This approach may be more generalizable to the broader EoE population, rather than just those who are hospitalized and may have more severe or complex disease. Furthermore, our study reveals that patients with EoE are more likely to progress to cirrhosis, an outcome not evaluated in the prior inpatient analysis. The alignment between our results and those of Kohli et al. supports the existence of a real-world link between EoE and liver disease, while differences in study design offer complementary perspectives on this emerging relationship.

The elevated risk of liver diseases risk in EoE is biologically plausible for attribution. EoE is a Th2-dominant condition marked by overproduction of interleukins such as IL-5, IL-13, and IL-4 in the esophageal tissue and peripheral circulation^4^. IL-4 may enhance hepatic de novo lipogenesis and promote steatosis, while IL-5 drives eosinophil proliferation and recruitment, and IL-13 contributes to fibrotic remodeling by activating fibroblasts and promoting collagen deposition 4, 20-22. Studies have shown that IL-13 is a potent pro-fibrogenic cytokine in the liver, capable of activating hepatic stellate cells and synergizing with TGF-β to exacerbate fibrosis^23^. In murine models of NASH, type 2 immunity exacerbated hepatic fibrosis, evidenced by higher IL-4/IL-13 levels and eosinophil accumulation in the livers of mice with Th2 polarization, whereas mice deficient in IL-4/IL-10 were protected from NASH progression^11^. Eosinophils traffic to the liver via CCR3/CCL11 signaling and release profibrotic mediators, including TGF-β, which could stimulate stellate-cell activation and fibrosis^24^. Barrier dysfunction in EoE, driven by IL-13-mediated down-regulation of filaggrin and tight-junction proteins^4^, ^25^, may extend to the intestine, permitting endotoxin translocation that fuels hepatic inflammation. These theoretical attributions suggest that a systemic Th2-inclined immune environment presenting in patients with active EoE, could tilt the balance toward hepatic inflammation and scarring if fatty liver is present.

Microbiome and Gut-Liver Axis and EoE treatments could also mediate the EoE-NAFLD association, as the gastrointestinal microbiome is a pivotal regulator of metabolism and inflammation. NAFLD has been strongly linked to gut microbiome alterations that increase intestinal permeability and the translocation of endotoxin into the portal circulation, triggering hepatic Toll-like receptor pathways and inflammation^26^. Patients with EoE have been found to exhibit a distinct microbiome in the esophagus and possibly the gut. Some studies suggest that EoE is associated with imbalanced microbial diversity and an overrepresentation of certain bacteria such as Haemophilus in the esophagus^27^. Additionally, chronic corticosteroid therapy and proton pump inhibitor use, both staples of EoE management, could exacerbate insulin resistance and microbial dysbiosis^28^-^30^, respectively, thereby amplifying NAFLD progression.

In sex-stratified analyses, female patients with EoE exhibited a relatively higher hazard ratio for incident NAFLD compared with male patients. A similar pattern was observed for cirrhosis, although statistical significance varied across subgroups. These findings suggest potential sex-specific modulation of the EoE-liver disease association. Sex hormones are known to influence both immune responses and hepatic fibrogenesis^31^. Estrogen has complex immunomodulatory properties and may interact with Th2-predominant pathways, while androgen signaling has been implicated in metabolic regulation and steatosis susceptibility^32^, ^33^. Although our study was not designed to establish mechanistic causality, these findings highlight the importance of considering sex as a potential biological variable in future investigations of immune-metabolic interactions in EoE.

This study possesses strengths as well as limitations. Chief among its strengths is the large-scale, multicenter, longitudinal design, which allowed for a comprehensive evaluation of temporal associations between EoE and hepatic outcomes. However, limitation should be concerned. First, as a retrospective observational cohort, the study is inherently limited in its ability to establish causality. Although we adjusted for several key metabolic risk factors, residual confounding remains possible due to unmeasured variables such as dietary patterns, physical activity, socioeconomic status, or concurrent medications. Medication-related confounding represents an important consideration in interpreting our findings. Proton pump inhibitors and corticosteroids are commonly prescribed in patients with EoE and have been independently associated with metabolic dysfunction and NAFLD risk in prior literature. Although the association between EoE and NAFLD persisted after excluding individuals exposed to these medications, effect estimates for cirrhosis attenuated and lost statistical significance, suggesting that medication exposure may partially contribute to advanced liver outcomes. Moreover, treatment-proxy definitions of EoE yielded higher hazard ratios, which may reflect greater disease severity, increased healthcare contact, or pharmacologic effects rather than a true biological dose-response relationship. Additionally, detailed data on medication adherence, cumulative dose, treatment duration, and over-the-counter proton pump inhibitor use were not available within the federated database. As such, time-varying exposure and precise quantification of medication effects could not be fully accounted for. These limitations should be considered when interpreting the magnitude of association, particularly for cirrhosis outcomes. Second, selection bias may be present, given that EoE cases were identified at tertiary care centers, potentially enriching for more severe phenotypes, while surveillance bias may have led to differential detection of NAFLD due to more frequent healthcare encounters among EoE patients. While we attempted to mitigate these biases through matching, their complete elimination is unlikely. Diagnostic misclassification is also a concern, as NAFLD and cirrhosis were identified through clinical documentation and imaging rather than systematic liver biopsy, and some controls may have had undiagnosed EoE. Third, the modest number of cirrhosis events limited statistical power for that outcome, and the lack of longitudinal histologic or elastographic liver assessments restricts insight into subclinical progression. Fourth, although body mass index was included in the matching process and stratification analyses, detailed measures of obesity severity such as visceral adiposity were not uniformly available within the federated database. While our BMI-stratified analyses demonstrated that the association between EoE and NAFLD persisted across both overweight and obese categories, residual confounding related to central adiposity cannot be entirely excluded. Notably, the magnitude of association for liver cirrhosis appeared greater among individuals with BMI ≥30 kg/m², raising the possibility that severe metabolic dysfunction may amplify the pro-fibrotic effects of systemic type 2 inflammation. Fifth, while our follow-up duration was substantial, it may not fully capture the long latency of NAFLD-related liver disease. Despite these limitations, the study provides important hypothesis-generating evidence of a potential link between EoE and adverse hepatic outcomes, highlighting the need for prospective, mechanistic, and interventional studies to further elucidate this relationship.

In summary, this retrospective cohort study demonstrates that adults diagnosed with EoE are at an elevated risk for developing NAFLD and liver cirrhosis compared with matched controls, with these associations remaining robust across various sensitivity analyses. These results underscore the importance of incorporating hepatic concerns into the clinical management of EoE and suggest that shared immune-metabolic mechanisms may serve as novel therapeutic targets.

## Supplementary Material

Supplementary tables.

## Figures and Tables

**Table 1:** Baseline characteristics

Covariates	Before matching				After matching^a^		
	EoE cohort (n= 44,759)	Control cohort (n= 6,094,112)	SMD		EoE cohort (n=44,758)	Control cohort (n=44,758)	SMD
**Age at index**							
Mean ± SD	32.4 ± 17.3	37.5 ± 20.3	0.27		32.4 ± 17.3	32.4 ± 17.4	0.00
**Sex**							
Male	25712 (57.4)	2515569 (41.4)	0.33		25712 (57.4)	26058 (58.2)	0.02
Female	16669 (37.2)	3175851 (52.2)	0.30		16669 (37.2)	16623 (37.1)	0.00
Unknown Gender	2377 (5.3)	391387 (6.4)	0.05		2377 (5.3)	2077 (4.6)	0.03
**Race, n (%)**							
White	35193 (78.6)	3646131 (59.9)	0.41		35193 (78.6)	35202 (78.7)	0.00
Black or African American	2198 (4.9)	797136 (13.1)	0.29		2198 (4.9)	2953 (6.6)	0.07
Asian	623 (1.4)	276187 (4.5)	0.19		623 (1.4)	933 (2.1)	0.05
American Indian or Alaska Native	154 (0.3)	28365 (0.5)	0.02		154 (0.3)	120 (0.3)	0.01
Native Hawaiian or Other Pacific Islander	165 (0.4)	51455 (0.8)	0.06		165 (0.4)	187 (0.4)	0.01
Other Race	1033 (2.3)	186945 (3.1)	0.05		1033 (2.3)	768 (1.7)	0.04
Unknown Race	5392 (12.0)	1096588 (18.0)	0.17		5392 (12.0)	4595 (10.3)	0.06
**Socioeconomic status**							
Persons with potential health hazards related to socioeconomic and psychosocial circumstances	688 (1.5)	92722 (1.5)	0.00		688 (1.5)	667 (1.5)	0.00
**Lifestyle**							
Mental and behavioral disorders due to psychoactive substance use	2375 (5.3)	380544 (6.3)	0.04		2375 (5.3)	2378 (5.3)	0.00
**Medical Utilization**							
Visit: Ambulatory	30289 (67.7)	3455391 (56.8)	0.23		30289 (67.7)	30278 (67.6)	0.00
Visit: Inpatient Encounter	6027 (13.5)	805855 (13.2)	0.01		6027 (13.5)	5989 (13.4)	0.00
**BMI**							
Greater than 25 kg/m^2^	13906 (31.1)	1551683 (25.5)	0.12		13906 (31.1)	13936 (31.1)	0.00
**Comorbidities**							
Essential hypertension	4184 (9.3)	831801 (13.7)	0.14		4184 (9.3)	4191 (9.4)	0.00
Hyperlipidemia	2819 (6.3)	510544 (8.4)	0.08		2819 (6.3)	2805 (6.3)	0.00
Diabetes mellitus	1368 (3.1)	337881 (5.6)	0.12		1368 (3.1)	1373 (3.1)	0.00
Major depressive disorder	1023 (2.3)	107967 (1.8)	0.04		1023 (2.3)	854 (1.9)	0.03
Chronic ischemic heart disease	654 (1.5)	159153 (2.6)	0.08		654 (1.5)	639 (1.4)	0.00
Chronic kidney disease	469 (1.0)	100570 (1.7)	0.05		469 (1.0)	454 (1.0)	0.00

EoE, eosinophilic esophagitis; SMD, standardized mean difference; SD, Standardized difference. ^a^ Propensity score matching 1:1 on age at index, sex, race, body mass index, medical utilization status, status of comorbidities (diabetes mellitus, hypertension, hyperlipidemia, chronic kidney disease, chronic ischemic heart disease), substance abuse (mental and behavioral disorders due to psychoactive substance use), socioeconomic status
