# Supplementary material for: Eosinophilic Esophagitis and Risk of Non-Alcoholic Fatty Liver Disease and Cirrhosis: A Multi-Center Retrospective Study
Source: Int J Med Sci. 2026 Jul 13;23(8):2670–8. doi: 10.7150/ijms.123768 (PMC13411181; doi:10.7150/ijms.123768)
Supplement: Supplementary file 1 — Supplementary tables. [file ijmsv23p2670s1.pdf]

Supplementary Files

Table S1. Utilized proxy codes [a]

**Description**	**ICD-10-CM codes**
Study population	
Eosinophilic esophagitis	ICD-10-CM K20.0
Outcome events	
Non-alcoholic fatty liver disease	ICD-10-CM: K75.81, K76.0
Liver cirrhosis or fibrosis	ICD-10-CM: K74
Covariates and other definitions	
Essential hypertension	ICD-10-CM: I10
Hyperlipidemia	ICD-10-CM: E78.5
Diabetes mellitus	ICD-10-CM: E08-E15
Major depressive disorder	ICD-10-CM: F33
Chronic ischemic heart disease	ICD-10-CM: I25
Chronic kidney disease	ICD-10-CM: N18
Persons with potential health hazards related to socioeconomic and psychosocial circumstances	ICD-10-CM: Z55-Z65
Mental and behavioral disorders due to psychoactive substance use	ICD-10-CM: F10-F19
Encounter for general examination	ICD-10-CM: Z00
Comedications	
Corticosteroids for systemic use	ATC code: H02
HMG-CoA reductase inhibitors	ATC code: C10AA

Glucagon-like peptide-1 analogues	ATC code: A10BJ

[a]ICD-10-CM: International Classification of Diseases, Tenth Revision, Clinical Modification; ATC code: Anatomical Therapeutic Chemical Classification System code

Table S2. Description of all applied sensitivity analysis models in Figure 1

**Models**	**Description**
**Applying various proxy-based eosinophilic esophagitis (EoE) definition**	
Algorithm 1	Only patients with the record of ICD-10-CM codes of K20.0 (eosinophilic esophagitis) with prescription record of proton pump inhibitors (ATC code: A02BC) were included as EoE group in this model.
Algorithm 2	Only patients with the record of ICD-10-CM codes of K20.0 (eosinophilic esophagitis) with prescription record of corticosteroids (ATC code: R01AD) were included as EoE group in this model.
Algorithm 3	Only patients with the record of ICD-10-CM codes of K20.0 (eosinophilic esophagitis) with greater than 2 instances of esophagogastroduodenoscopy procedures (CPT code: 1021431) were included as EoE group in this model.
**Applying different wash-out period after index date**	
12 months/24 months after index date	Any outcome events occurring during the designated washout period were excluded from subsequent analyses. For all subgroup analyses, the follow-up duration was standardized to 15 years.
5 years /10 years /15 years after index date	Only outcome events occurring within the defined follow-up period were included in the analysis. In all subgroup analyses, a washout period of 3 months was applied.
**Applying different matching covariates**	
Crude model	The hazard ratio was estimated using the population prior to the application of propensity score matching.
Matching model 1	Matching covariates include age at index and sex

Matching model 2	Matching covariates include age at index, sex, socioeconomic status and medical utilization status
